# Rethinking the Obesity Paradox: BMI and Longitudinal Cognitive Decline in Older Adults

**DOI:** 10.1002/alz70861_108137

**Published:** 2025-12-23

**Authors:** Subhamoy Pal, Bilal Irfan, Jonathan M Reader, Kelly M. Bakulski, Voyko Kavcic, Bruno Giordani

**Affiliations:** ^1^ Michigan Alzheimer's Disease Research Center, Ann Arbor, MI USA; ^2^ University of Michigan, Ann Arbor, MI USA; ^3^ Wayne State University, Detroit, MI USA; ^4^ University of Michigan Medical School, Ann Arbor, MI USA

## Abstract

**Background:**

Higher body mass index (BMI) has often been linked to a heightened risk for cognitive decline; however, emerging evidence suggests a potential “obesity paradox,” in which overweight or obese older adults may actually experience a slower rate of cognitive decline. Using a large, longitudinal cohort of older adults from 25 sites across the United States, we examined the relationship between BMI and cognition.

**Method:**

Data were drawn from the National Alzheimer’s Coordinating Center (NACC) database over five annual visits. Cognition was measured by the Montreal Cognitive Assessment (MoCA). Clinically‐assessed BMI was categorized as normal, overweight, and obese. Time‐varying covariate measures included age and presence of clinically‐assessed comorbidities (i.e., diabetes, hyperlipidemia, and hypertension). To test the association between MoCA and BMI, we used linear‐mixed effects regression models with random intercepts and fixed effects for BMI, time, and their interaction with covariate measures. We performed sensitivity analyses stratified by age (55‐75, and ≥ 76).

**Result:**

In the full sample (*n* =526 participants, *p* =2630 observations), total raw MoCA scores significantly decreased from visit 1 to visit 5 (β = ‐0.71, *p*<0.05). Age was negatively associated with MoCA (β = ‐0.09, *p*<0.5). There was no significant main effect of BMI on MoCA, however there were significant interactions between BMI categories and time. Specifically, obese (β = 0.86, *p*< 0.05) and overweight (β = 0.95, *p* <0.05) participants showed a slower rate of decline in MoCA over five visits compared to normal‐weight individuals. The same pattern of results held for participants aged 55‐75 as in the full sample. However, among those 76 years and older, being overweight or obese no longer significantly reduced the MoCA decline over the five visits. Comorbidities were not associated with MoCA in any of the models.

**Conclusion:**

In contrast to some previous research, our findings suggest a complex, and at times contradictory, relationship between weight status and MoCA score, where overweight or obesity may modestly buffer against cognitive decline based on age. Further research should explore potential age‐dependent effects, clarify casual pathways, and determine whether targeted interventions around BMI might modify late‐life cognitive trajectories.

